# The safety and efficacy of gamma knife surgery in management of glomus jugulare tumor

**DOI:** 10.1186/1477-7819-8-76

**Published:** 2010-09-06

**Authors:** Raef FA Hafez, Magad S Morgan, Osama M Fahmy

**Affiliations:** 1 Neurosurgery and Gamma knife department, International Medical Center, Cairo, Egypt

## Abstract

**Background:**

Glomus jugulare is a slowly growing, locally destructive tumor located in the skull base with difficult surgical access. The operative approach is, complicated by the fact that lesions may be both intra and extradural with engulfment of critical neurovascular structures. The tumor is frequently highly vascular, thus tumor resection entails a great deal of morbidity and not infrequent mortality. At timeslarge residual tumors are left behind. To decrease the morbidity associated with surgical resection of glomus jugulare, gamma knife surgery (GKS) was performed as an alternative in 13 patients to evaluate its safety and efficacy.

**Methods:**

A retrospective review of 13 residual or unresectable glomus jagulare treated with GKS between 2004 and 2008.. Of these, 11 patients underwent GKS as the primary management and one case each was treated for postoperative residual disease and postembolization. The radiosurgical dose to the tumor margin ranged between 12-15 Gy.

**Results:**

Post- gamma knife surgery and during the follow-up period twelve patients demonstrated neurological stability while clinical improvement was achieved in 5 patients. One case developed transient partial 7th nerve palsy that responded to medical treatment. In all patients radiographic MRI follow-up was obtained, the tumor size decreased in two cases and remained stable (local tumor control) in eleven patients.

**Conclusions:**

Gamma knife surgery provids tumor control with a lowering of risk of developing a new cranial nerve injury in early follow-up period. This procedure can be safely used as a primary management tool in patients with glomus jugulare tumors, or in patients with recurrent tumors in this location. If long-term results with GKS are equally effective it will emerge as a good alternative to surgical resection.

## Introduction

Glomus jugulare tumors are rare, slow-growing, hypervascular tumors that arise within the jugular foramen of the temporal bone. They are included in a group of tumors referred as paragangliomas, which occur at various sites and include carotid body, glomus vagale, and glomus tympanic tumors. These tumors frequently invade the adjacent jugular bulb, internal carotid artery and the lower cranial nerves The occurrence is reported in a ratio of 1:1,000,000 in the fifth to sixth decade of life [1].

Glomus jugulare tumors are locally destructive lesions located in one of the poorly accessible surgical regions of the skull base. The operative approach is, complicated by the fact that lesions may be both intradural and extradural with engulfment of critical neurovascular structures. Thus, it is not surprising that resection entails a great deal of morbidity, and not infrequent mortality at times leaving behind large residual tumors [2-5].

Time to diagnosis from the first symptom is range between four and six years. Thus by the time they are presented to a surgeon the tumors are often very large and are unlikely to be resected completely. Treatment is controversial. Traditional treatment options include surgery with or without preoperative embolization followed by postoperative conventional external beam radiotherapy. These have been associated with significant morbidity and mortality [6-9].

Glomus jugulare tumors occur predominantly in women in the fifth and sixth decades of life. Because of the insidious onset of symptoms, these tumors often go unnoticed, and delay in diagnosis is frequent. Because of the location and extent of involvement, glomus jugulare tumors present a significant diagnostic and management challenge. Although rare, glomus jagulare is the most common tumor of the middle ear and are second to vestibular schwannoma as the most common tumor of the temporal bone [10].

The most common symptoms are conductive hearing loss and pulsatile tinnitus. Other aural signs and symptoms are ear fullness, otorrhea, hemorrhage, bruit, and the presence of a middle ear mass. Significant ear pain is uncommon. Involvement of the inner ear produces vertigo and sensorineural hearing loss [11].

Cranial nerve involvement produces hoarseness and dysphagia. The presence of jugular foramen syndrome (paresis of cranial nerves IX-XI) is pathognomonic of this tumor, but it usually follows the initial symptoms of hearing loss and pulsatile tinnitus. Less commonly, glomus tumors produce facial nerve palsy, hypoglossal nerve palsy, or horner syndrome. Ataxia and brain stem symptoms may also develop. Involvement of the dural sinuses may mimic sinus thrombosis [11,12].

In about 2-4% of cases, the first or leading symptoms are hypertension and tachycardia (pheochromocytoma like symptoms) produced by catecholamines, norepinephrine, or dopamine excreted by the tumor. Also, somatostatin, vasoactive intestinal polypeptide, calcitonin, and neuron-specific enolase may be produced by the tumor. Other related symptoms include headache, perspiration, pallor, and nausea [13,14].

The treatment of glomus jugulare tumors presents the surgeon with a significant management problem. Because the neoplasm originates in the region of the jugular bulb, it frequently involves the lower cranial nerves, with occasional extension into the posterior fossa. Despite extensive work on the development of surgical and radiation treatment strategies, considerable controversy still exists regarding the optimal management of these lesions. Despite these therapies, tumor control can be difficult to achieve particularly without undue risk of patient morbidity or mortality [15].

Microsurgical removal of glomus jugulare tumors is frequently associated with injury of the lower cranial nerves. To decrease the morbidity associated with tumor management, gamma knife surgery (GKS) has been performed as an alternative to resection [16].

Traditionally, conventional fractionated external beam radiotherapy was used to treat residual tumors with varying degrees of success ranging from a maximum of 61-71% to an average of 23%. Side-effects include osteoradionecrosis of the temporal bone, radiation necrosis of the temporal lobe, mastoiditis and second malignancies [10]. Although the glomus cells *per se *are radioresistant and radiotherapy helps to halt tumor growth by inducing fibrosis around the supplying vessels. Stereotactic radiosurgery with the Gamma knife system delivers precise high-dose radiation to a small localized field to increase the chances of obliterative endarteritis while reducing complications by sparing adjacent normal structures [7,17].

## Materials and methods

### Objective

To evaluate the safety and efficacy of gamma knife surgery (GKS) for controlling the glomus jugulare tumors

### Method

Between 2004 and 2008, 13 cases with glomus jagulare tumors were treated using gamma knife surgery at the International Medical Center, Cairo, Egypt. The follow-up period ranged from 12 to 48 months. All patients underwent a complete neurological assessment before the treatment that included MRI and audiograms. Follow-up included clinical neurological evaluation and MRI brain that were done regularly at 6 monthly intervals in the first year and yearly afterward.

### Radiosurgery technique

The Elekta Leksell^® ^gamma knife was used for the treatment. Target localization was achieved using MRI performed with T1 axial and coronal-weighted sequence at 2 mm slice thickness with and without contrast, T1- fat saturation sequence and also T2 axial sequence was used to eliminate tumor edema. Treatment planning was performed with Elekta Leksell^® ^Gamma Plan. Treatment peripheral dose ranged between 12-15 Gy usually at 35% to 50% isodose curve. The maximum dose to the adjacent brain stem area ranged between 10 - 12 Gy.

## Results

Eleven patients underwent gamma knife surgery as primary treatment, one had partial microsurgical tumor removal and one had underwent tumor embolization pre-gamma knife surgery.

**Figure 1:**
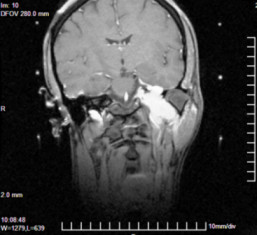
**Stereotactic MRI + contrast pre-gamma knife surgery for glomus jugulare tumor extends to the atlas vertebrae level**.

The mean age of patients was 43.6 years (range, 22-64 years). There were 11 females and two male. The tumors were located at left side in 10 cases and right side in 3 cases (Figure 1). The most common neurological deficit was IX, X, XI cranial nerve paresis in 7 patients, sensorineural deterioration in hearing, facial paresis, XII cranial nerve paresis and trigeminal impairment were also recorded. Pulstile tinnitus was recorded in 9 cases and ataxia in 3 cases.

**Figure 2:**
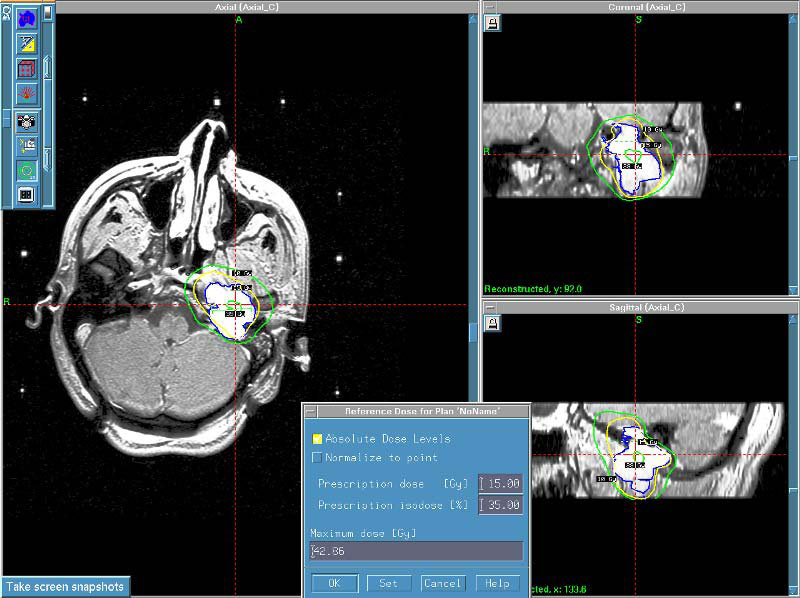
**Gamma plan for the same case of the glomus jugulare treated with 15 Gy to the margin at 35% isodose curve**.

Of the 13 tumors that underwent GKS, the mean tumor volume was 8.4 cc (range 2.6-19.4 cc). The tumor peripheral dose was 15 Gy in all cases at mean isodose curve of 37.7% (range 35 to 50%), (Figure 2).

The clinical follow-up period ranged between 12 to 48 months. All patients had follow up clinically and by MRI at 6 monthly interval in the first year and yearly afterward.

Clinically the improvement was detected in 5 cases during the follow-up period (starting in 6 months to 24 months post gamma knife surgery). Improvement was mainly in dysphonia, regurgitation and also shoulder pain. Seven cases showed stable clinical disease with no additional symptoms or signs.

One patient developed transient partial 7th nerve palsy at 9 months post gamma knife surgery.

**Figure 3:**
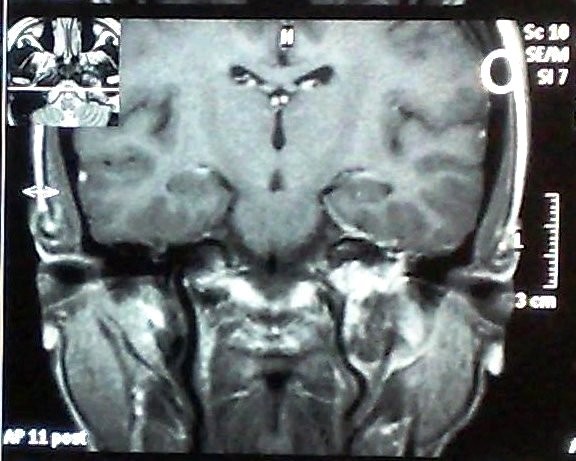
**36 months post gamma knife surgery follow-up MRI with contrast for the same case showed loss of central tumor enhancement with reduction of the treated glomus jugulare tumor**.

Magnetic resonance imaging follow-up was available for all the 13 patients. Eleven patients showed local tumor control and two patients showed decrease in tumor size (Figure 3).

## Discussion

Glomus jagulare tumors though radioresistant, radiation has been found to be helpful in controlling tumor growth by inducing fibrosis around the supplying vessels [7,11,13].

In a study by Pollock (2004) GKS was used as the primary management in 19 patients and for recurrent glomus jugulare tumors for 23 patients.Of these, 12 tumors (31%) decreased in size, 26 (67%) remained unchanged, and one (2%) grew. The patient whose tumor grew underwent repeated GKS. Progression-free survival after GKS was 100% at 3 and 7 years, and 75% at 10 years. Six patients (15%) experienced new deficits (hearing loss alone in three, facial numbness and hearing loss in one, vocal cord paralysis and hearing loss in one, and temporary imbalance and/or vertigo in one). In 26 patients in whom hearing could be tested before GKS, hearing preservation was achieved in 86% and 81% at 1 and 4 years post treatment, respectively [14].

Ganj and Abdelkarim [8] reported on 14 patients with glomus jagulare tumorstreated with mean dose of 13.6 Gy (range 12-16) with mean follow-up period of 28 months (range 6 to 60 months). All the tumors except one were Fisch type D and the mean volume was 14.2 cm^3^, (range 3.7-28.4 cm^3^). Volume of eight lesions became smaller while 6 remain unchanged. Two patients with bruit had no improvement in their symptoms. Among the other 12, 5 had symptomatic improvement of dysphagia, 4 in dysphonia, 3 in facial numbness and 3 in ataxia [8].

In our study, 5 patients showed improvement in their neurological symptoms and seven cases had stable clinical disease. Radiologically Eleven patients showed local tumor control in the follow-up MRI and two patients showed decrease in tumor size. Clincial improvement was seen irrespective of the tumor response.

Stereotactic radiosurgery with the Gamma knife system delivers precise high-dose radiation to a small localized field to increase the chances of obliterative endarteritis while reducing complications by sparing adjacent normal structures. With the present results the GKS appears to be a viable alternative for large, residual or recurrent glomus juglare tumors. Longer follow-up periods are required to assess long-term effects in a benign disease, tumor control and quality of life indices would appear to be more significant than eradication [11,12].

## Conclusion

Gamma Knife Surgery is a safe and effective treatment for glomus jugulare tumors, particularly in patients with preserved glossopharyngeal and vagus nerve function, after surgical recurrence, in the elderly, and in patients with serious preexisting medical conditions.

## Competing interests

The authors declare that they have no competing interests.

## Authors' contributions

RFAH conceived and prepared the manuscript. MSM and OMF participated in the design of the study. All authors read and approved the final manuscript.
